# Comprehensive pan-cancer analysis reveals the prognostic value and immunological role of SPIB

**DOI:** 10.18632/aging.204225

**Published:** 2022-08-14

**Authors:** Meng Ding, Qiufang Li, Xiao Tan, Liangyua Zhang, Jun Tan, Lan Zheng

**Affiliations:** 1 Key Laboratory of Physical Fitness and Exercise Rehabilitation of Hunan Province, Hunan Normal University, Changsha, China

**Keywords:** pan-cancer, bioinformatics, prognosis, immune infiltration, TMB, MSI

## Abstract

It is well-established that SPIB is essential for the survival of mature B cells, playing a key role in diffuse large B-cell lymphoma, colorectal cancer, and lung cancer. However, no study has hitherto conducted a systematic pan-cancer analysis on SPIB. Herein, we analyzed the differential expression of SPIB in pan-cancer using The Cancer Genome Atlas (TCGA) and Genotype-Tissue Expression (GTEx) databases and found that SPIB was significantly upregulated in most cancers. In addition, SPIB was positively or negatively associated with prognosis in different cancers. We found that SPIB was significantly associated with tumor immune infiltration and immune checkpoint genes in more than 35 tumors by TIMER database analysis. In addition, SPIB was negatively correlated with Tumor mutational burden (TMB) and Microsatellite instability (MSI) in most tumors. Finally, GO/KEGG enrichment analysis revealed the possible involvement of SPIB in NF-kappa B and B-cell receptor signaling pathways. In conclusion, our comprehensive pan-cancer analysis of SPIB reveals its important role in tumor immunity, suggesting it has huge prospects for clinical application in cancer therapy.

## INTRODUCTION

Current evidence suggests that cancer incidence is increasing, associated with increased burden on society [1]. Despite the clinical success of existing treatments (surgery, radiotherapy, chemotherapy, immunotherapy, and targeted therapy), patient prognosis and survival rates remain low [1, 2]. Therefore, the quest for new tumor markers for the diagnosis and treatment of cancer is essential.

SPIB is an ETS transcription factor involved in B cell receptor-mediated signal transduction [3]. SPIB is expressed in pDCs, CD34+ precursor cells, and mature B cells [4, 5] and is associated with the differentiation of plasmacytoid dendritic cells and intestinal microfold cells [6, 7]. An increasing body of evidence from recently published studies suggests that SPIB plays an important role in tumor development. For instance, SPIB reportedly exerts an inhibitory effect in colorectal cancer cells by activating NFkB and JNK signaling through MAP4K1 [8] and promotes tumor-associated macrophage (TAM) recruitment by enhancing the expression of CCL4 in lung cancer [9]. Moreover, SPIB plays an important role in the abnormal switch reorganization of ABC DLBCL [10]. Overwhelming evidence from tissue microarray-based studies has linked SPIB to ovarian cancer, liver cancer, esophageal squamous cell carcinoma, head and neck squamous cell carcinoma, gastric cancer, and hepatocellular carcinoma [11–16]. Immunotherapy has emerged as a new pillar of cancer treatment, mediating tumor regression by blocking immune checkpoints [17]. Although SPIB has been studied in more than 9 tumors, most studies have focused only on specific cancer types. Therefore, a deeper understanding of the relationship between SPIB and the immune microenvironment of different tumors is essential to provide the basis for exploring new immune-related therapeutic targets and clinical treatment of cancer.

In this study, we comprehensively analyzed the prognostic relationship between SPIB expression levels and various cancers based on data mining analysis of various databases, including The Cancer Genome Atlas (TCGA), Genotype Tissue-Expression (GTEx), Human Protein Atlas (HPA), Tumor Immune Estimation Resource (TIMER), and cBioPortal. We also explored the role of SPIB in the immune response to further visualize its prognostic outlook in pan-cancer. Our comprehensive analysis revealed that SPIB has prognostic value in various cancers and plays an important role in tumor immunity by affecting tumor-infiltrating immune cells, TMB and MSI. In addition, potential signaling pathways of SPIB were identified by gene set enrichment analysis (GSEA). In conclusion, we systematically and comprehensively investigated SPIB expression in pan-cancer to screen cancer types with poor prognosis and provide the foothold for future studies.

## MATERIALS AND METHODS

### Data sources and gene transcription expression analysis

We obtained the pan-cancer dataset TCGA TARGET GTEx (PANCAN, *N* = 19131, G = 60499) from the University of California Santa Cruz (UCSC) Xena browser (https://xenabrowser.net/) [18]. The expression data of SPIB in each sample was extracted, and log2(x + 0.001) transformed. Finally, tumor types with less than 3 samples were excluded, and the expression data of 34 tumors were obtained (See Supplementary Table 1 for further details). Differences in expression between normal and tumor samples for each tumor type were calculated, and significant differences were analyzed using unpaired Wilcoxon Rank Sum and Signed Rank Tests.

### Genetic alteration analysis

Data on genetic alterations (Mutation, Amplification, Deep Deletion) of SPIB in 32 tumors were obtained through the cBioPortal database [19]. The cancer type summary module in the cBioPortal database was used to obtain summary graphs of Mutation, Amplification, and Deep Deletion of genes in TCGA tumors.

### Protein level analysis

The protein expression levels of SPIB in normal and tumor tissues were analyzed in the Human Protein Atlas (HPA, https://www.proteinatlas.org/) database. The SPIB protein-protein interaction network (PPI) was constructed using the String (https://string-db.org/) database.

### Survival prognosis analysis

Univariate Cox regression analysis was performed using the R package survival (version 3.2-7) [20]. The coxph function was used to establish a Cox proportional hazards regression model and analyze the prognostic relationship of SPIB in various tumors, while the significance of differences was assessed by the Log-rank test. The analytical data are shown in Supplementary Table 2.

### Immune infiltration and immune checkpoint analysis

The correlation between SPIB expression and the level of immune cell infiltration was analyzed using the gene expression module of the TIMER2.0 database (http://timer.comp-genomics.org/) [21]. Immune cell types included B cell, CD4 T cell, CD8 T cell, neutrophil, macrophage, and dendritic cell (DC); the analysis results are shown in Supplementary Table 3. Spearman's correlation analysis was used to assess the relationship between SPIB expression and expression levels of immune checkpoint markers. Sixty markers were identified, including inhibitory (*n* = 24) and stimulatory (*n* = 36) markers. In addition, we analyzed the relationship between SPIB expression and immune scores, including StromalScore, ImmuneScore, and ESTIMATEScore. Similarly, scatter plots were used to show the top 6 tumors with significant differences, respectively (Supplementary Table 4).

### Co-expression analysis of immune regulatory genes

Co-expression analysis of SPIB and immunomodulatory genes, including chemokine (41), receptor (18), MHC (21), Immunoinhibitor (24), and Immunostimulator (46) was performed using the R package limma. The co-expression data are shown in Supplementary Table 5.

### Tumor mutation burden and microsatellite instability analysis

The dataset for calculating TMB comes from Simple Nucleotide Variation at level 4 of all TCGA samples processed by MuTect2 software (DOI: 10.1038/nature08822). The MSI score for each tumor was derived from a previous study [22]. The TMB of each tumor was calculated using the TMB function of the R package maftools (version 2.8.05). The relationship between SAPIB expression and TMB and MSI was analyzed using the Pearson correlation coefficient.

### Gene enrichment analysis

Using the GO annotations of genes in the R package org.Hs.eg.db (version 3.1.0) as the background, the genes were mapped to the background set, and gene set enrichment analysis was conducted by the R package clusterProfiler (version 3.14.3). The minimum number of gene sets was set to 5 and the maximum to 5000; *P*-value of <0.05 and FDR of <0.1 were considered statistically significant. The detailed analysis results are shown in Supplementary Table 6. In addition, we extracted the top 100 SPIB-related genes in TCGA by GEPIA2 and analyzed the expression correlation between SPIB and the top 5 targeted genes.

### Statistical analysis

Correlations between variables were analyzed using Pearson or Spearman analysis. Prognostic survival curves were generated using Kaplan-Meier analysis while applying the log-rank test to estimate statistical significance. The significance level was set at *P* < 0.05. All statistical data analyses were performed using R software version 3.6.4.

### Data availability statement

All data generated or analyzed during this study are included in this published article (and its Supplementary Information files).

## RESULTS

### SPIB expression is upregulated in most cancers

**Figure 1:**
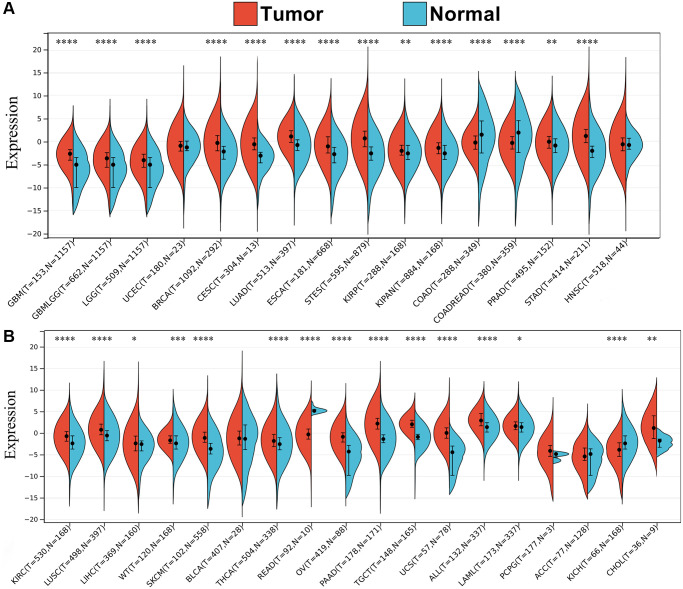
**Pan-cancer expression profiling of SPIB.** (**A**, **B**) Differential expression of SPIB in 34 cancers based on TCGA and GTEx databases, blue represents normal tissues, and red represents tumor tissues. ^*^P < 0.05, ^**^P < 0.01, ^***^P < 0.001, ^****^P < 0.0001. *P*-value *P* < 0.05 was considered statistically significant.

To clarify whether SPIB is associated with cancer, we analyzed the mRNA expression of SPIB in normal and tumor tissues using TCGA and GTEx databases. The results showed that SPIB expression was significantly upregulated in 25 out of 33 cancer types compared to adjacent normal tissues, including GBM, GBMLGG, LGG, BRCA, CESC, LUAD, ESCA, STES, KIRP, KIPAN, PRAD, STAD, KIRC, LUSC, LIHC, WT, SKCM, THCA, OV, PAAD, TGCT, UCS, ALL, LAML and CHOL (Figure 1A, 1B). In contrast, SPIB was significantly downregulated in COAD, COADREAD, READ, and KICH (Figure 1A, 1B). These results suggest that SPIB may function as an oncogenic molecule in various cancers. Although gene mutation is not a sufficient condition for carcinogenesis, carcinogenesis depends on the accumulation of gene mutations. Therefore, examining genetic alterations associated with the SPIB gene in cancer patients is essential. We performed a comparative analysis of SPIB using the cBioPortal database and found that SPIB amplification was one of the most important single alterations in Mature B-Cell Neoplasms, ACC, BRCA, BLCA, PAAD, and LIHC (Supplementary Figure 1). In addition, SPIB exhibited the highest mutation frequency in ESCA, UCEC, SKCM, Non-Seminomatous Germ Cell Tumors, COAD, HNSC, Non-Small Cell Lung Cancer, and SARC (Supplementary Figure 1).

**Figure 2:**
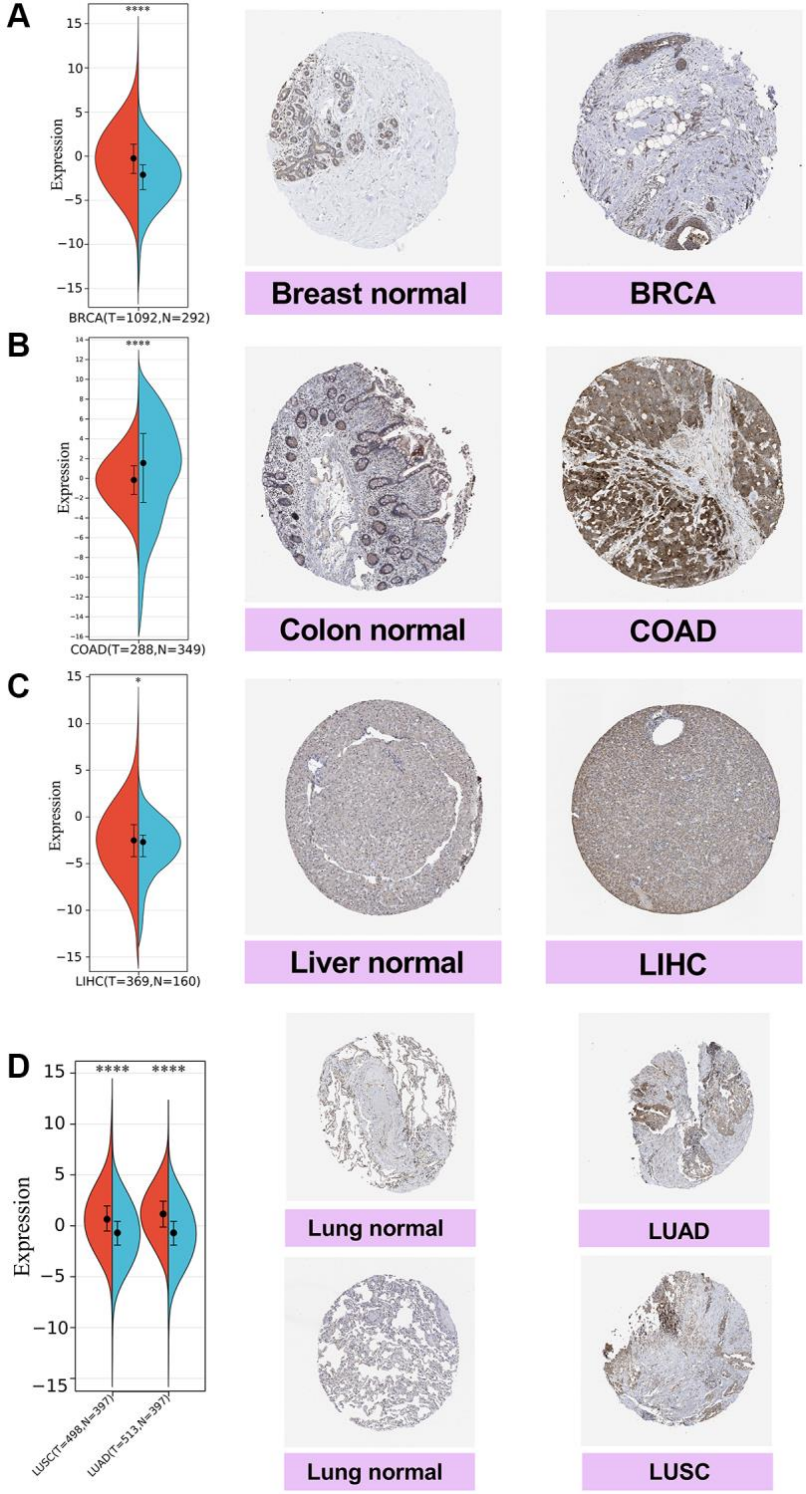
**Comparison of SPIB expression in breast, colon, liver, and lung tissues by immunohistochemistry.** (**A**) Breast tissue. (**B**) Colon tissue. (**C**) Liver tissue. (**D**) Lung tissue, including lung adenocarcinoma (LUAD) and lung squamous carcinoma (LUSC). For the violin plot, red is cancer samples and blue is normal samples. ^*^P < 0.05, ^**^P < 0.01, ^***^P < 0.001, ^****^P < 0.0001.

Next, we compared the SPIB gene expression from the TCGA database with the Immunohistochemistry (IHC) results from the HPA database. The results showed negative SPIB expression in normal breast and liver tissues, while tumor tissues showed moderate IHC staining (Figure 2A, 2C). SPIB staining was weak in normal colon tissues, while tumor tissues exhibited strong IHC staining (Figure 2B). In addition, SPIB staining was weak in normal lung tissue, while tumor tissue exhibited moderate staining (Figure 2D). Taken together, these results illustrate the consistency of the TCGA and HPA database results.

### The correlation between SPIB and the prognosis for different tumor types

**Figure 3:**
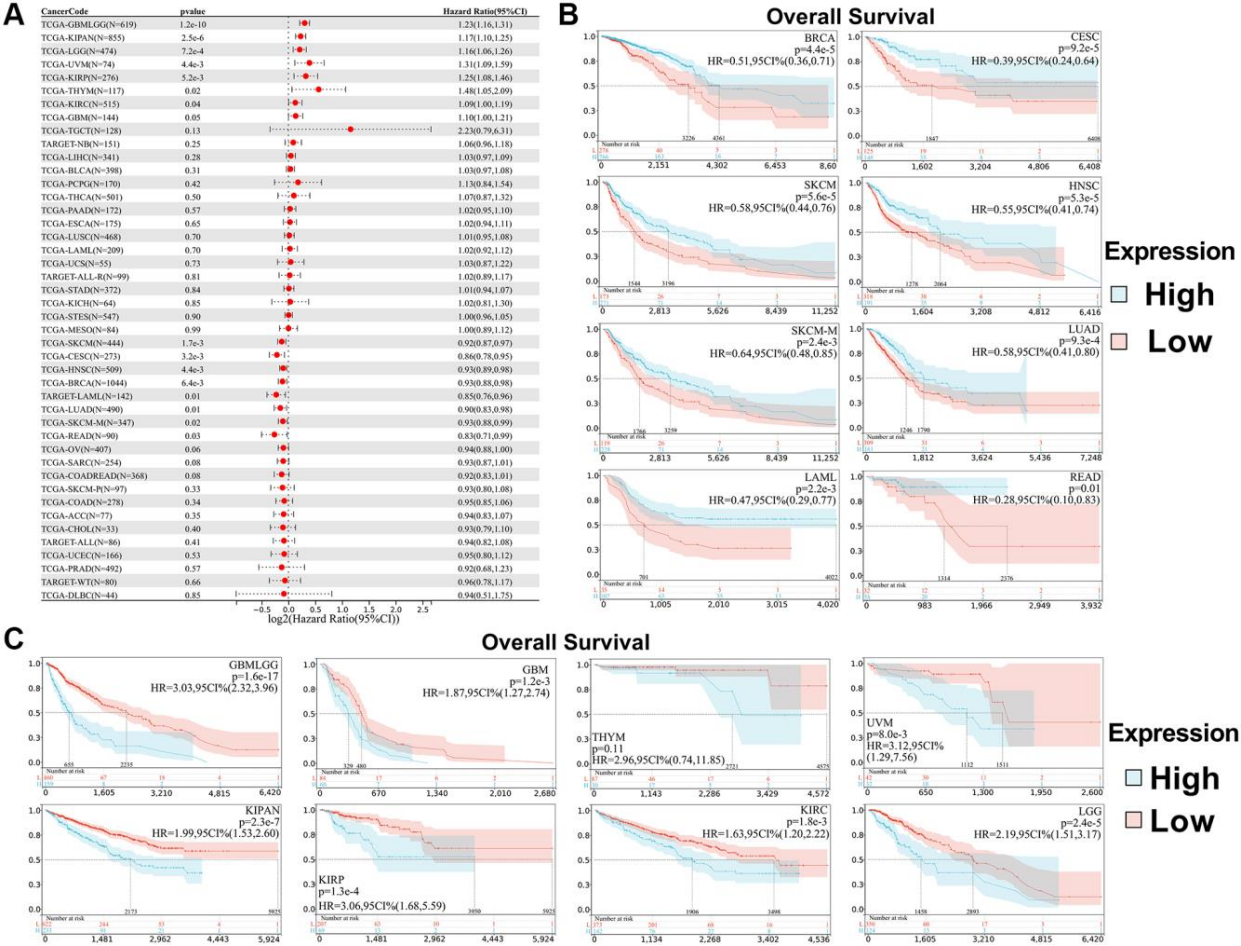
**Relationship between SPIB expression and overall survival (OS) in pan-cancer.** (**A**) Cox regression analysis of SPIB in 44 tumors. (**B**) Kaplan-Meier OS curves of SPIB expression in patients with LAML, BRCA, CESC, LUAD, HNSC, SKCM, SKCM-M, and READ. (**C**) Kaplan-Meier OS curves of SPIB expression in patients with GBMLGG, LGG, KIRP, KIPAN, GBM, KIRC, THYM, and UVM. For (**B** and **C**), the vertical coordinate is the survival probability, and the horizontal coordinate is the survival time (days).

We used the coxph function of the R package surv (version 3.2-7) to build a Cox proportional hazards regression model, analyze the prognostic relationship between SPIB expression and prognosis in each tumor, and obtain prognostic significance using a statistical test by Log-rank test [20]. The results showed that SPIB expression affected overall survival (OS) in patients with 16 cancer types, including GBMLGG, LGG, KIRP, KIPAN, GBM, KIRC, THYM, UVM, LAML, BRCA, CESC, LUAD, HNSC, SKCM, SKCM-M, and READ (Figure 3A). In addition, Kaplan-Meier survival analysis showed that reduced SPIB expression correlated with poor OS in LAML, BRCA, CESC, LUAD, HNSC, SKCM, SKCM-M, and READ (Figure 3B). Meanwhile, increased SPIB expression correlated with a poor OS in GBMLGG, LGG, KIRP, KIPAN, GBM, KIRC, THYM, and UVM (Figure 3C).

**Figure 4:**
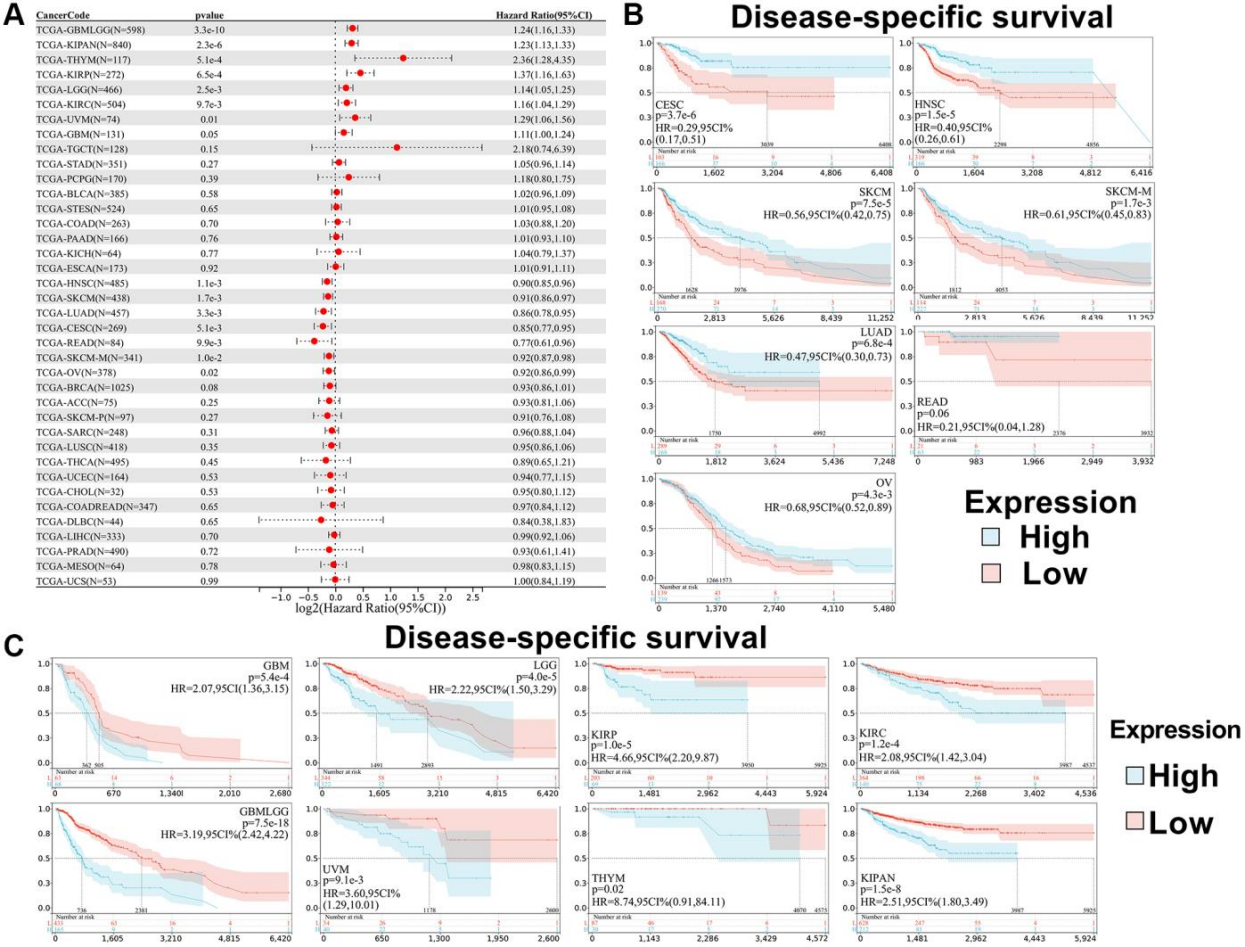
**Relationship between SPIB expression and disease-specific survival (DSS) in pan-cancer.** (**A**) Cox regression analysis of SPIB in 44 tumors. (**B**) Kaplan-Meier OS curves of SPIB expression in patients with CESC, LUAD, HNSC, SKCM, SKCM-M, READ, and OV. (**C**) Kaplan-Meier OS curves of SPIB expression in patients with GBMLGG, LGG, KIRP, KIPAN, GBM, KIRC, THYM, and UVM. For (**B** and **C**), the vertical coordinate is the survival probability, and the horizontal coordinate is the survival time (days).

In addition, we analyzed the correlation between SPIB expression and patient DSS. The results showed that SPIB expression affected Disease-Specific Survival (DSS) in 15 cancer types, including GBMLGG, LGG, KIRP, KIPAN, GBM, KIRC, THYM, UVM, CESC, LUAD, HNSC, SKCM, SKCM-M, READ, and OV (Figure 4A). Kaplan-Meier analysis indicated that decreased SPIB expression correlated with a poor prognosis in CESC, LUAD, HNSC, SKCM, SKCM-M, READ, and OV, whereas increased SPIB expression was associated with poor prognosis in GBMLGG, LGG, KIRP, KIPAN, GBM, KIRC, THYM, and UVM (Figure 4B, 4C).

**Figure 5:**
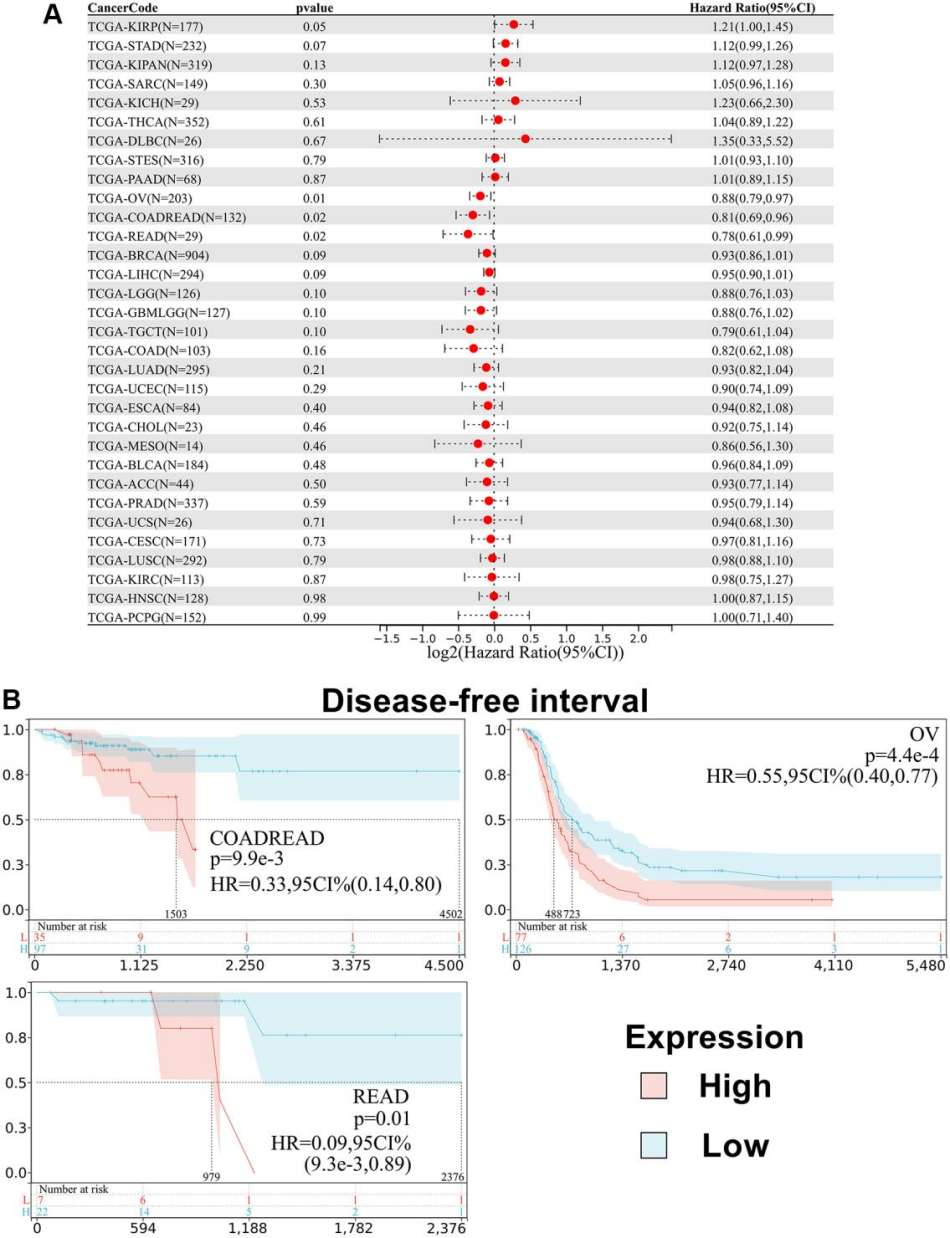
**Relationship between SPIB expression and disease-free interval (DFI) in pan-cancer.** (**A**) Cox regression analysis of SPIB in 44 tumors. (**B**) Kaplan-Meier OS curves of SPIB expression in patients with COADREAD, OV, and READ. The vertical coordinate is the survival probability, and the horizontal coordinate is the survival time (days).

Besides, we analyzed the relationship between SPIB expression and patient Disease-Free Interval (DFI). The results showed that SPIB expression affected DFI in patients with 3 cancer types, including COADREAD, OV, and READ (Figure 5A). Kaplan-Meier analysis showed that reduced SPIB expression correlated with a poor prognosis in COADREAD, OV, and READ (Figure 5B).

Finally, we analyzed the correlation between SPIB expression and patient Progression-Free Interval (PFI). The results showed that SPIB expression affected PFI in patients with 12 cancer types, including GBMLGG, LGG, KIRP, KIPAN, GBM, UVM, BRCA, LUAD, HNSC, SKCM, SKCM-M, and OV (Supplementary Figure 2A). Kaplan-Meier analysis showed that reduced SPIB expression in GBMLGG, LGG, KIRP, KIPAN, GBM, and UVM correlated with a poor prognosis, whereas increased SPIB expression was associated with poor prognosis in BRCA, LUAD, HNSC, SKCM, SKCM-M, and OV (Supplementary Figure 2B, 2C). In conclusion, these data suggest that SPIB expression determines the prognosis of patients with different types of cancers, in terms of OS, DSS, DFI or PFI.

### High SPIB expression is associated with immune infiltration of multiple tumors

**Figure 6:**
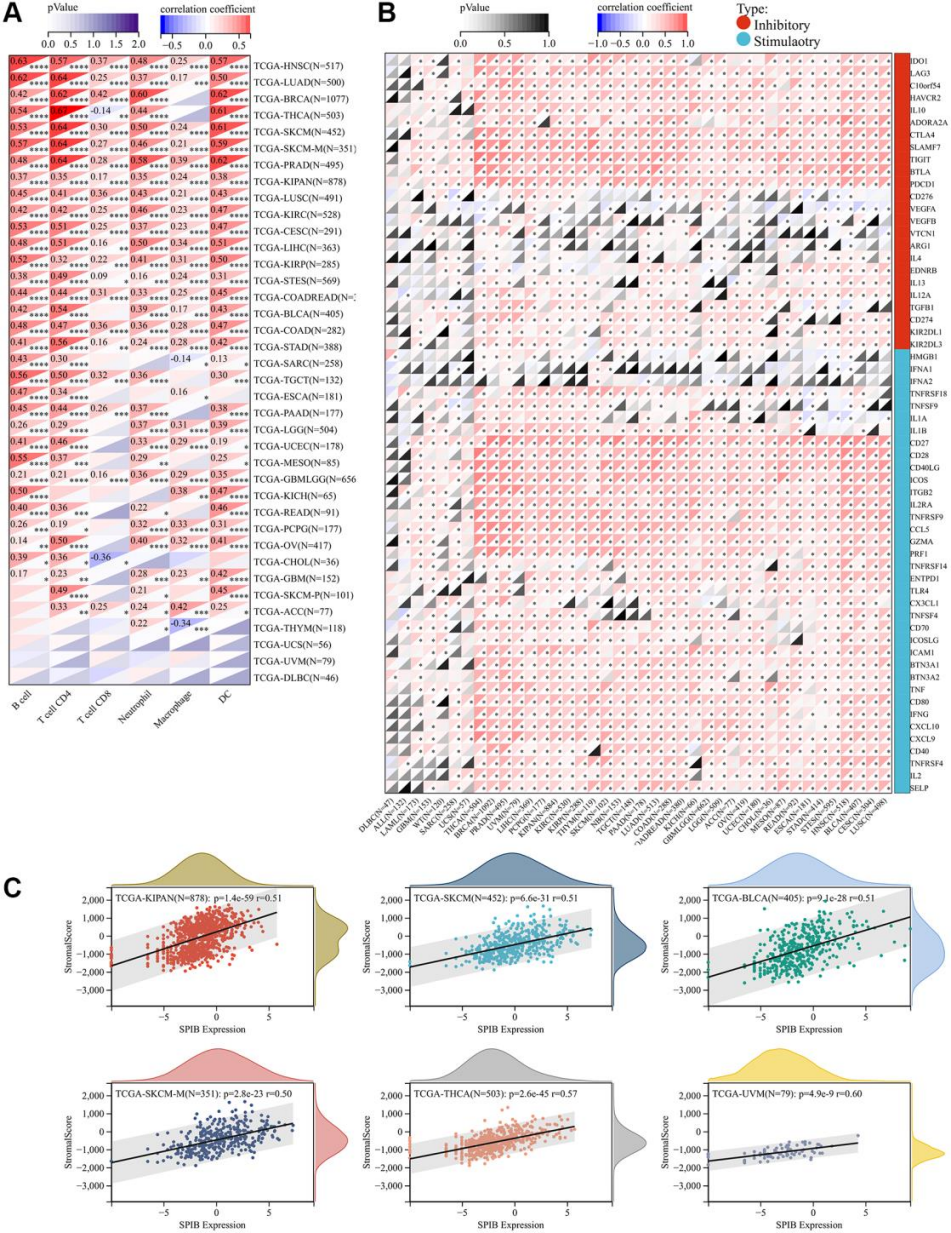
**SPIB expression correlates with immune infiltration and immune checkpoint markers.** (**A**) SPIB expression and infiltration levels of various immune cells in the TIMER database. (**B**) Correlation analysis of SPIB expression levels with the levels of 60 immune checkpoint genes in pan-cancer. (**C**) SPIB expression in UVM, THCA, KIPAN, SKCM, BLCA, and SKCM-M immune infiltration levels. ^*^P < 0.05, ^**^P < 0.01, ^***^P < 0.001, ^****^P < 0.0001.

To elucidate the association between SPIB expression and specific immune cell types in pan-cancer, we evaluated the correlation between SPIB expression and immune cell infiltration in pan-cancer based on the TIMER database. We obtained six types of immune cell infiltration scores for 9,406 tumor samples from 38 cancer types and found that high SPIB expression was significantly associated with immune infiltration in 35 tumors (Figure 6A). SPIB expression was associated with B cell, CD4 T cell, CD8 T cell, neutrophil, macrophage, and DC in 32, 33, 20, 31, 25, and 32 tumors, respectively (Figure 6A). In addition, we selected 60 immune checkpoint genes for analysis, including inhibitory (*n* = 24) and stimulatory (*n* = 36) genes. Interestingly, the results of immune checkpoint analysis showed that SPIB expression was positively correlated with most immune checkpoint genes (Figure 6B). In this respect, more than 50 immune checkpoint genes were positively correlated with SPIB expression in LIHC, KIPAN, PRAD, OV, THCA, BRCA, COADREAD, LUAD, BLCA, HNSC, PAAD, and COAD (Figure 6B). In addition, in DLBC, the expression of SPIB was least correlated with the immune checkpoint genes (Figure 6B). To further assess the role of SPIB in the tumor immune microenvironment, we analyzed the relationship between SPIB expression and immune infiltration score in tumors using ESTIMATE. The most significant positive correlation between SPIB expression and immune infiltration was found in UVM, THCA, KIPAN, SKCM, BLCA, and SKCM-M (Figure 6C).

In addition, we analyzed the relationship between SPIB expression and immune-related genes in different tumors, including chemokines, chemokine receptors, MHC, immunosuppression, and immune activation. The results showed that most immune-related genes were co-expressed with SPIB (Supplementary Figure 3). Interestingly, most tumors were positively correlated with SPIB except for ALL, DLBC, and UCS (Supplementary Figure 3). Overall, our results suggest that SPIB plays a key role in immune infiltration and immune escape in most tumors.

### SPIB expression negatively correlates with TMB and MSI in a variety of tumors

**Figure 7:**
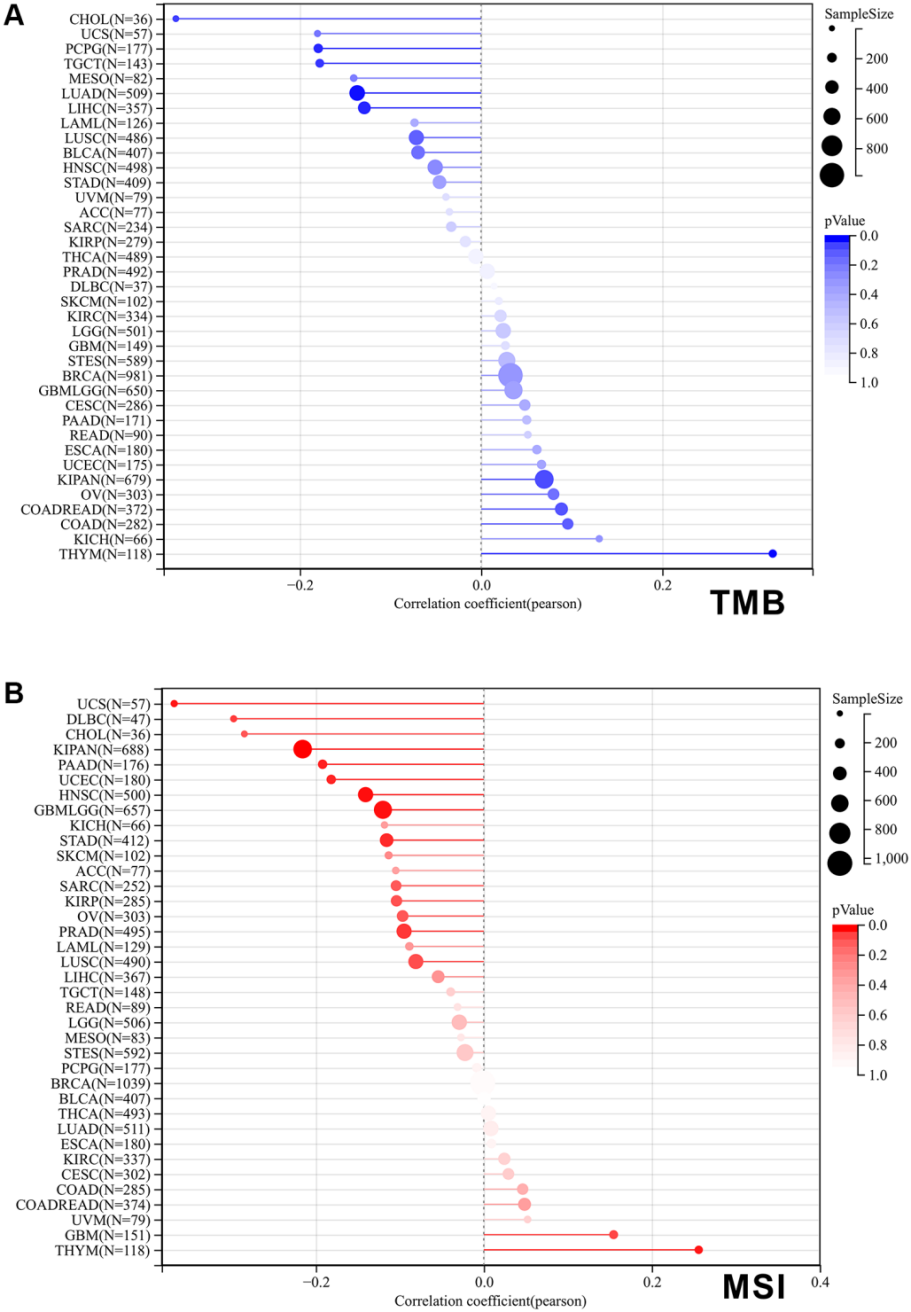
**Correlation of SPIB expression with tumor mutational load (TMB) and microsatellite instability (MSI) in pan-cancer.** (**A**) The bar graph represents the correlation between SPIB expression and TMB in pan-cancer. (**B**) The bar graph represents the correlation between SPIB expression and MSI in pan-cancer. *P* values are from Spearman's correlation analysis.

Tumor Mutational Burden (TMB) and Microsatellite Instability (MSI) are well-established as key markers for immunotherapy [23, 24]. Therefore, we evaluated the correlation of SPIB expression with TMB and MSI. The results showed that SPIB expression was correlated positively with TMB of THYM and negatively with TMB of LUAD, LIHC, TGCT, PCPG, and CHOL (Figure 7A). In addition, SPIB expression correlated positively with the MSI of THYM and negatively with the TMB of GBMLGG, KIPAN, STAD, PRAD, UCEC, HNSC, PAAD, UCS, and DLBC (Figure 7B).

### SPIB-related genes mainly mediate immune-related pathways

**Figure 8:**
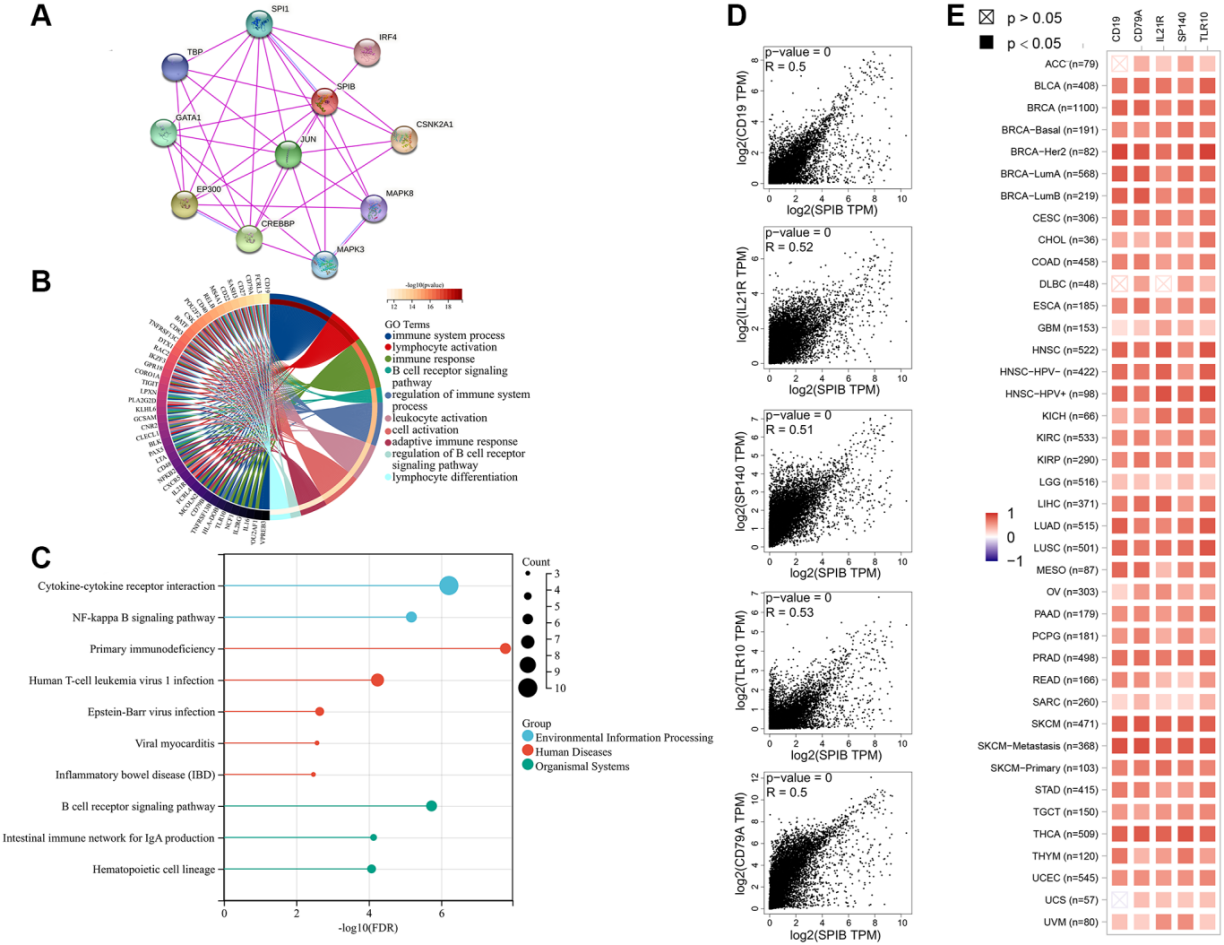
**SPIB-related gene enrichment and pathway analysis.** (**A**) SPIB protein network map based on STRING database. (**B**) GO analysis of the top 100 genes associated with SPIB expression. (**C**) KEGG analysis of the top 100 genes associated with SPIB expression. (**D**) Correlation between the most relevant genes for SPIB expression, including PEZ1, GNA12, MAP4, SEPT7 TPST1, and TBL2 of the GEPIA2 tool. (**E**) Heatmap of genes significantly associated with SPIB expression.

Finally, we investigated the molecular mechanisms underlying the occurrence of SPIB in tumors. We screened the proteins interacting with SPIB by STRING database and generated a PPI interaction network (Figure 8A). We found that 10 proteins were directly associated with SPIB. The top 100 genes related to SPIB expression were then obtained using the GEPIA database, and the five genes with the highest correlation were selected, including CD19, CD79A, IL21R, SP140, and TLR10 (Figure 8D). In addition, GO analysis suggested genes associated with SPIB expression were significantly enriched in immune system process, lymphocyte activation, immune response, B cell receptor signaling pathway, and regulation of immune system process (Figure 8B). KEGG analysis showed that genes associated with SPIB expression were enriched in Cytokine-cytokine receptor interaction, NF-kappa B signaling pathway, Primary immunodeficiency, Human T-cell leukemia virus 1 infection, and B cell receptor signaling pathway (Figure 8C). In addition, SPIB had a strong positive correlation with the above five genes in most cancer types (Figure 8E).

## DISCUSSION

SPIB plays a crucial role in the development of B cells [4]. There is a rich literature available suggesting that SPIB promotes trans-activation of SPI1 to increase glycolytic gene expression and drive the glycolytic process, proliferation, and invasiveness of colon cancer cells [25]. In addition, SPIB is a novel prognostic factor in DLBCL, mediating apoptosis through the PI3K-AKT pathway [26]. Over the years, many studies have linked SPIB to tumors, including lung, gastric, and colorectal cancers. Thus a comprehensive pan-cancer study of SPIB may present a novel perspective on other tumors.

Our study revealed significant differences in SPIB in 29 tumors, including high expression in 25 tumors and low expression in 4 tumors. We also analyzed the protein levels of SPIB expression in breast, colon, liver, and lung tissues by IHC experiments based on the HPA database and confirmed this trend. The importance of identifying tumor-specific targets or features is widely acknowledged, given that individualized treatment based on their characteristics can increase the chances of curing cancer [27]. A pan-cancer analysis is an effective method to discover differential expression and characteristics of tumors. Therefore, we analyzed the relationship between SPIB expression and prognosis in various tumors using TCGA and GTEx databases. In this study, we found that high SPIB expression correlated with poor OS and DSS in patients with GBMLGG, LGG, KIRP, KIPAN, GBM, KIRC, THYM, and UVM. Low SPIB expression was associated with a poor OS in patients with LAML, BRCA, CESC, LUAD, HNSC, SKCM, and SKCM-M, and poor DSS in patients with CESC, SKCM, and SKCM-M. These findings substantiate that SPIB may serve as a predictor of tumor prognosis. Although these survival analyses have clinical significance and provide a foothold for future studies, *in vivo* or *in vitro* validation experiments are lacking. Therefore, the role of SPIB in different types of cancer remains to be further investigated.

In recent years, immunotherapy has become a new pillar in treating tumors. The tumor immune microenvironment is an important component of the tumor microenvironment, and the mechanism by which tumor cells work with the immune microenvironment is important for selecting key molecules for tumor markers and potential drug targets [28, 29]. In addition, tumor-infiltrating lymphocytes, namely tumor-associated macrophages and tumor-infiltrating neutrophils (TIN), play an important role in tumor immunity [30, 31]. These findings corroborate that tumor-infiltrating immune cells play an important role in tumor progression, emphasizing the need to analyze the role of SPIB in the immune microenvironment. We found that SPIB expression was significantly associated with six immune infiltrating cells in most cancers, except UCS, UVM, and DLBC, consistent with previous studies that reported that therapeutic targeting of SPIB/SPI1 promotes the interaction of cancer cells and neutrophils to inhibit aerobic glycolysis and cancer progression [25]. Moreover, it has been shown that SPIB overexpression in mouse models increased infiltration of TAM, especially M2 macrophages, and promoted lung cancer progression [9]. In addition, immune checkpoint genes can directly affect immune cell function [32]. During tumorigenesis, immune checkpoint (IC) signaling is activated by tumors to undergo immune escape and accounts for tumor aggressiveness. Therefore, analyzing the correlation between SPIB expression and immune checkpoint markers could provide new targets for developing novel immunosuppressive agents. In this study, we analyzed more than 50 immune checkpoint genes. The results showed that SPIB expression was positively correlated with immune checkpoints in most tumors, except DLBC, ALL, LAML, and UCS. In addition, our study revealed that SPIB was co-expressed with genes encoding MHC, immune activation, immune suppression, chemokine, and chemokine receptor proteins. These results imply an immunological role for SPIB in various tumors and highlight its potential as a therapeutic target.

TMB is a more accurate and comprehensive potential biomarker. Current evidence suggests that TMB is associated with the occurrence of mutations and the synthesis of abnormal proteins that activate antitumor responses [33]. MSI is a predictive biomarker with potential significance for ICI responses. It has been shown that MSI leads to the accumulation of mutations that result in the formation of neoantigens and the activation of antitumor immune responses [34]. Our study showed that SPIB expression was associated with TMB and MS in THYM, LUAD, LIHC, TGCT, PCPG, CHOL, BMLGG, KIPAN, STAD, PRAD, UCEC, HNSC, PAAD, UCS, and DLBC. However, further experimental validation of the therapeutic role of SPIB in these cancers is warranted.

We also performed an enrichment analysis of genes related to SPIB expression. IRF4 and SPI1 identified in the SPIB protein interactions network are reportedly involved in B cell differentiation and play a critical and non-redundant role in the adaptive immune response of mature B cells [35, 36]. CREBBP and EP300 are associated with DLBCL, and CREBBP/EP300 mutations induce H3K27 deacetylation and activate the NOTCH signaling pathway, which is closely associated with B-cell malignancies [37]. In addition, the Jun protein family members are involved in the constitutive dimer AP-1. AP-1 activity is involved in various cellular processes; for instance, AP-1 plays a crucial role in several aspects of the immune system, such as T cell activation, T helper (Th) differentiation, T cell incompetence, and failure [38]. MAPKs regulate various cellular activities in cancer progression, including proliferation, apoptosis, and immune escape, and blocking upstream kinases is an important therapeutic strategy [39]. An increasing body of evidence suggests that TLP (TBP-2), a family member of TBP, is a tumor suppressor gene that plays a key role in DC-induced T-cell responses [40, 41]. GATA1 reportedly regulates the basal transcription of mSTING genes and plays a key role in the pathogenesis of autoimmune diseases and cancer [42]. CSNK2A1 is involved in tumorigenesis by phosphorylating various proteins, including SIRT1 [43]. In the present study, we also performed GO and KEGG analyses of genes related to SPIB expression. Overall, the results showed that SPIB was enriched in the immune system process, lymphocyte activation, immune response, B cell receptor signaling pathway, and regulation of the immune system process. These results are consistent with previous studies that SPIB is essential in protective humoral immunity [4]. In addition, SPIB has been associated with tumor suppression via NF-kappa B signaling pathway [8], consistent with our study findings.

In a nutshell, our study demonstrates an important role for SPIB in cancer that is not limited to specific cancer types. We investigated the relationship of SPIB in pan-cancer with prognosis, immune cell infiltration, tumor mutational load, and microsatellite instability and comprehensively assessed its potential as a prognostic biomarker from multiple perspectives. Despite our extensive analytical work, there are many limitations. First of all, the heterogeneity associated with different databases may affect the robustness of our analysis results to a certain extent. Besides, our results are based on public database analysis involving at least 33 cancer types, making experimental validation challenging. Although SPIB promotion of cancer progression has been experimentally validated in colon cancer cells and ABC-DLBCL [25, 44], more clinical or animal experiments are still needed to validate it. Nonetheless, our pan-cancer study analysis of SPIB still provides the basis and novel insights for future studies.

## Supplementary Materials

Supplementary Figures

Supplementary Table 1

Supplementary Table 2

Supplementary Table 3

Supplementary Table 4

Supplementary Table 5

Supplementary Table 6
