# Supplementary material for: Comprehensive pan-cancer analysis reveals the prognostic value and immunological role of SPIB
Source: Aging (Albany NY). 2022 Aug 14;14(15):6338–57. doi: 10.18632/aging.204225 (PMC9417235; doi:10.18632/aging.204225)
Supplement: Supplementary Figures [file aging-14-204225-s001.pdf]

SUPPLEMENTARY FIGURES

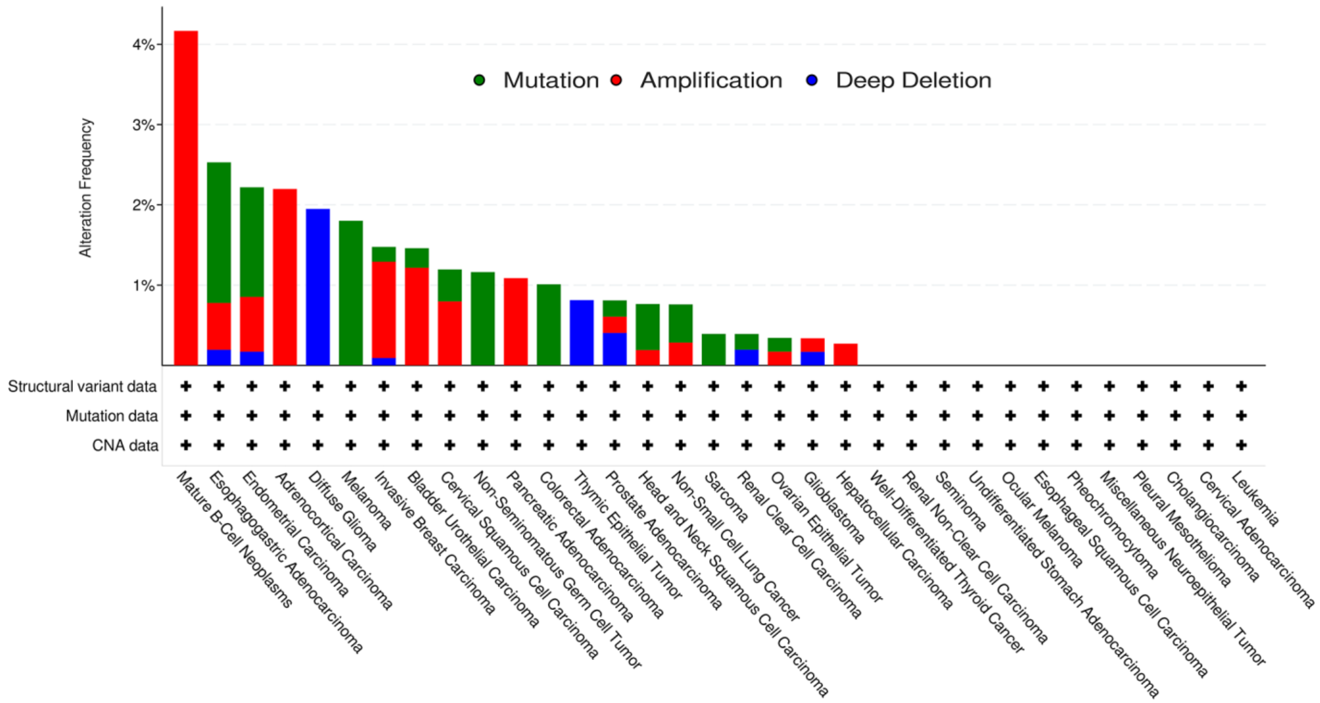

Supplementary Figure 1. Mutation of SPIB in pan-cancer. Analysis of genetic alteration type and mutation frequency of SPIB in different cancers based on the cBioPortal database.

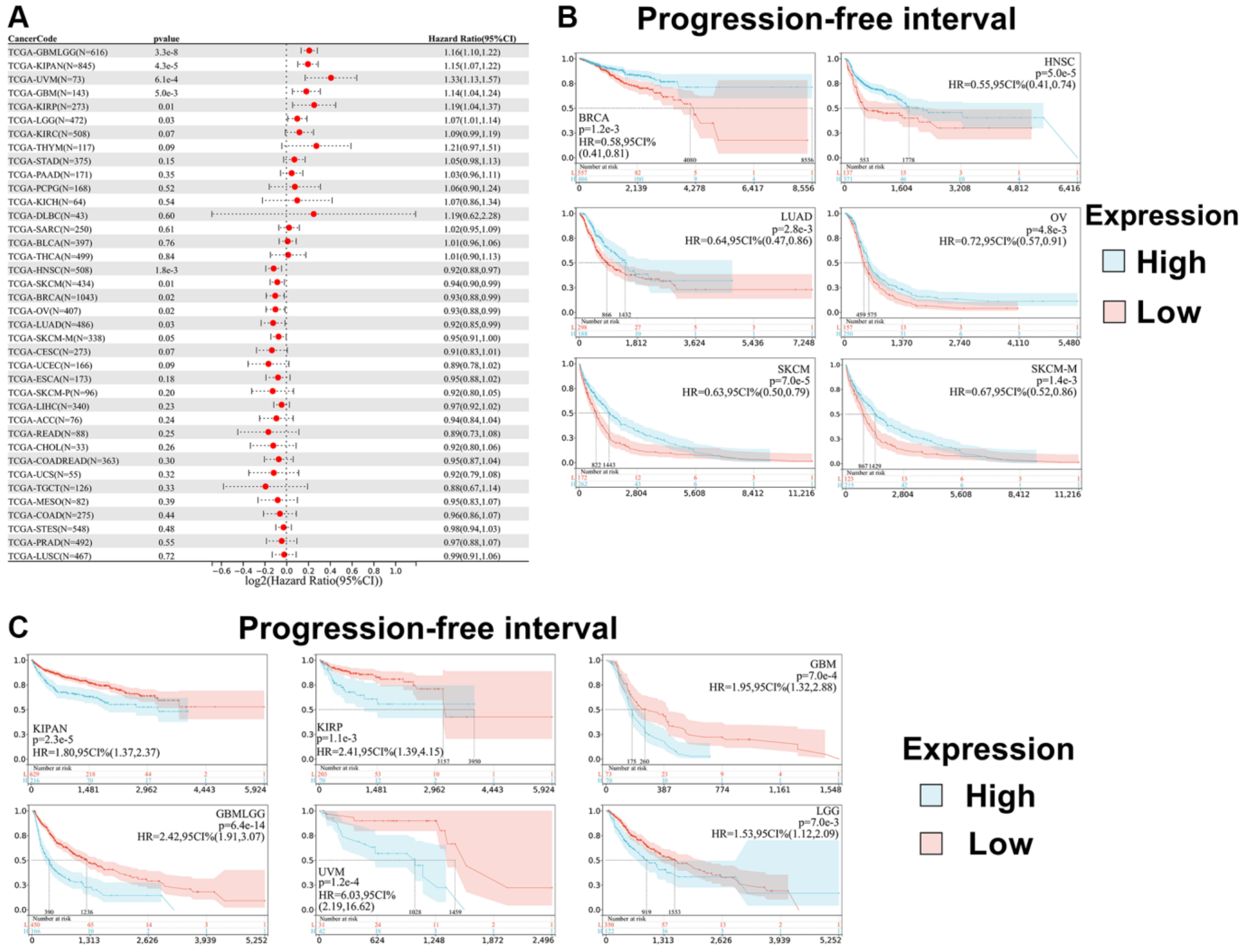

Supplementary Figure 2. Relationship between SPIB expression and progression-free interval (PFI) in pan-cancer. (**A**) Cox regression analysis of SPIB in 44 tumors. (**B**) Kaplan-Meier OS curves of SPIB expression in patients with GBMLGG, LGG, KIRP, KIPAN, GBM and UVM. (**C**) Kaplan-Meier OS curves of SPIB expression in patients with BRCA, LUAD, HNSC, SKCM, SKCM-M and OV. The vertical coordinate is the survival probability and the horizontal coordinate is the survival time (days).

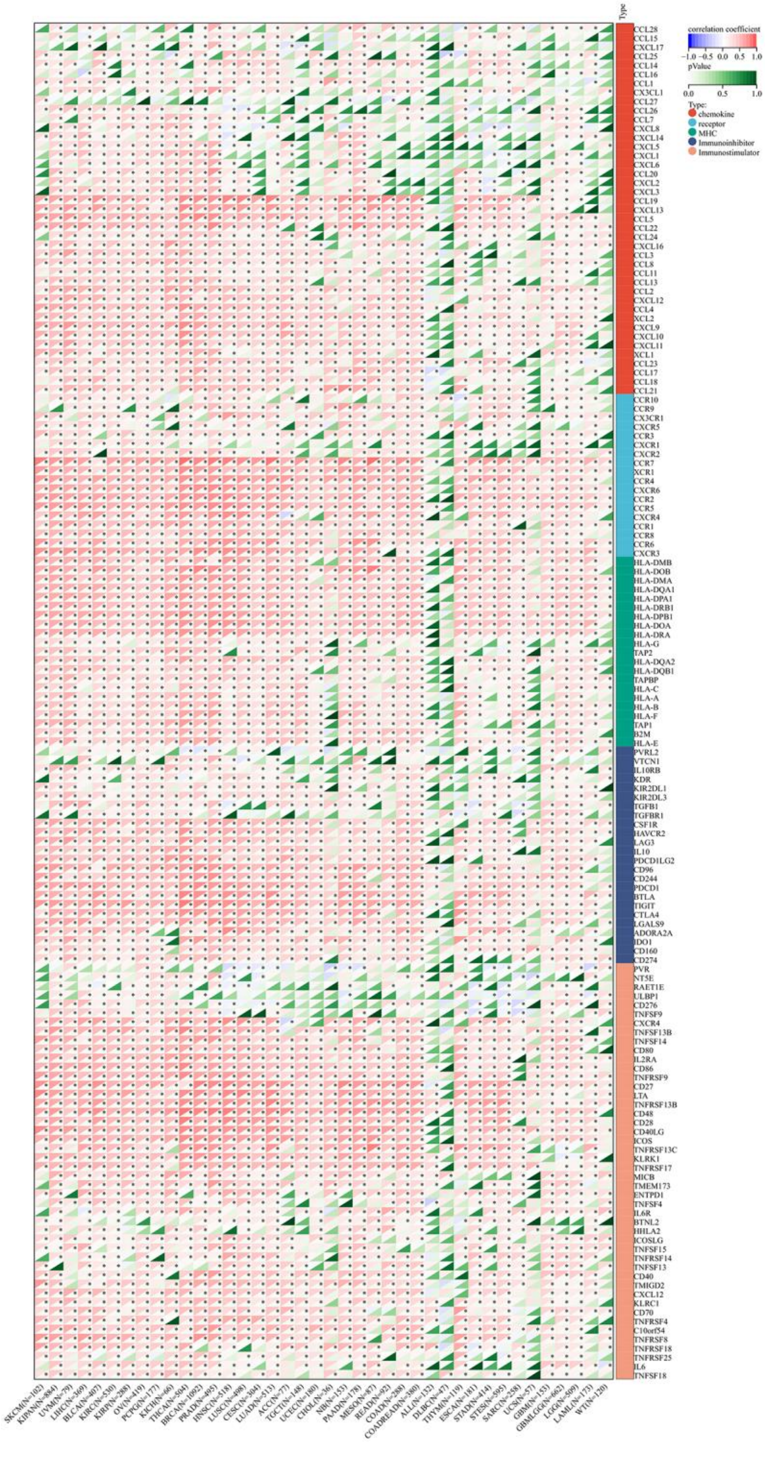

Supplementary Figure 3. Co-expression of TREM2 and immune-related genes, including five immune pathways chemokine (41), receptor (18), MHC (21), Immunoinhibitor (24), Immunostimulator (46) marker genes. $^{*}P < 0.05$, $^{**}P < 0.01$, $^{***}P < 0.001$, $^{****}P < 0.0001$.
