# Supplementary material for: Comprehensive pan-cancer analysis reveals the prognostic value and immunological role of SPIB
Source: Aging (Albany NY). 2022 Aug 14;14(15):6338–57. doi: 10.18632/aging.204225 (PMC9417235; doi:10.18632/aging.204225)
Supplement: Supplementary Table 5 [file aging-14-204225-s006.pdf]

Supplementary Table 5. Co-expression analysis of immune regulatory genes.

CODE	SampleNum	SigCount	PositiveCount	NegativeCount
SKCM	102	114	114	0
KIPAN	884	143	143	0
UVM	79	123	123	0
LIHC	369	142	139	3
BLCA	407	137	136	1
KIRC	530	139	139	0
KIRP	288	131	131	0
OV	419	146	146	0
PCPG	177	135	135	0
KICH	66	125	124	1
THCA	504	140	138	2
BRCA	1092	140	139	1
PRAD	495	143	142	1
HNSC	518	140	129	11
LUSC	498	130	126	4
CESC	304	127	123	4
LUAD	513	138	135	3
ACC	77	113	110	3
TGCT	148	111	110	1
UCEC	180	106	106	0
CHOL	36	67	66	1
NB	153	126	123	3
PAAD	178	137	136	1
MESO	87	102	102	0
READ	92	107	106	1
COAD	288	128	128	0
COADREAD	380	133	133	0
ALL	132	33	30	3
DLBC	47	11	10	1
THYM	119	112	107	5
ESCA	181	110	103	7
STAD	414	115	109	6
STES	595	130	119	11
SARC	258	99	91	8
UCS	57	37	36	1
GBM	153	115	115	0
GBMLGG	662	138	135	3
LGG	509	128	127	1
LAML	173	93	90	3
WT	120	58	57	1
